# Functional connectivity alterations in spinocerebellar ataxia type 10: insights from gray matter atrophy

**DOI:** 10.1007/s11682-026-01091-4

**Published:** 2026-02-07

**Authors:** Gustavo Padron-Rivera, Gabriel Ramirez‐Garcia, Amanda Chirino‐Perez, Angel Omar Romero-Molina, Adriana Ochoa-Morales, María Guadalupe Garcia-Gomar, Miguel Angel Ramirez‐Garcia, Omar Rodriguez-Mendoza, Diana Laura Torres-Vences, Birgitt Schüle, Erick Humberto Pasaye-Alcaraz, Carlos Roberto Hernandez-Castillo, Juan Fernandez‐Ruiz

**Affiliations:** 1 Laboratorio de Neuropsicologia, Departamento de Fisiologia, Facultad de Medicina, Universidad Nacional Autonoma de Mexico, Ciudad de Mexico, Mexico; 2 Departamento de Neurogenetica, Instituto Nacional de Neurologia y Neurocirugia Manuel Velasco Suarez, Ciudad de Mexico, Mexico; 3 Escuela Nacional de Estudios Superiores (ENES), Universidad Nacional Autónoma de México, Juriquilla Queretaro, Mexico; 4 Department Pathology, Stanford University School of Medicine, Stanford, CA USA; 5 Unidad de Resonancia Magnetica, Instituto de Neurobiología, Universidad Nacional Autónoma de México, Juriquilla Queretaro, Mexico; 6 Faculty of Computer Science, Dalhousie University, Halifax, Canada; 7 Instituto de Neuroetologia, Universidad Veracruzana, Xalapa Veracruz, Mexico; 8 Escuela Superior de Tlahuelilpan, Universidad Autonoma del Estado de Hidalgo, Pachuca, Mexico; 9 Department of Psychology, University of California, Berkeley, CA 94704 USA

**Keywords:** Spinocerebellar ataxia, Functional connectivity, Gray matter atrophy, Cerebellum, Cognition.

## Abstract

Spinocerebellar ataxia type 10 (SCA10) is a rare, inherited neurological disease caused by an expansion of the non-coding ATTCT pentanucleotide repeat in the ATAXIN 10 gene. It is characterized by cerebellar ataxia and epilepsy. Previous research has demonstrated extensive white and gray matter degeneration, particularly in the cerebellum. However, the impact of the SCA10 mutation on functional connectivity (FC) remains unexplored. This study aimed to characterize intrinsic FC changes in SCA10 patients and their relationship to clinical manifestations. Structural and resting-state Magnetic Resonance Imaging (MRI) were obtained from 26 SCA10 patients and 26 control subjects. Voxel-based morphometry (VBM) and seed-ROI and Independent Components Analysis (ICA) were performed to identify cerebral atrophy and FC changes respectively. Additionally, correlation analyses were conducted between FC changes and scores from the Scale for the Assessment and Rating of Ataxia (SARA) and the Montreal Cognitive Assessment (MoCA). In SCA10 patients, VBM analysis revealed extensive gray matter loss in motor cortices and the cerebellum. FC analysis identified significant FC changes originating from seed-ROIs in the right cerebellar VI and left precentral gyrus. Furthermore, group comparison using ICA components showed that SCA10 patients exhibited higher FC in the sensorimotor and cerebellar functional networks. Moreover, the average Blood Oxygen Level Dependent (BOLD) signal within the cerebellar network negatively correlated with MoCA scores. In summary, SCA10 patients exhibited enhanced FC in brain regions that displayed gray matter atrophy, underscoring the impact of SCA10 degeneration on resting state networks and induction of potential maladaptive FC compensatory mechanisms.

**Supplementary Information:**

The online version contains supplementary material available at 10.1007/s11682-026-01091-4.

## Introduction

Spinocerebellar ataxia type 10 (SCA10) is an autosomal dominant disease characterized by the co-occurrence of cerebellar ataxia and epilepsy. This complex clinical profile arises from the expansion of a pentanucleotide repeat sequence (ATTCT) within the ATXN10 gene (Matsuura et al., 2000). In addition to its core features, SCA10 is associated with a range of additional symptoms, including polyneuropathy, pyramidal signs, dysarthria, as well as cognitive deficits and psychiatric symptoms (Gatto et al., 2007; Teive & Arruda, 2009). Understanding the full clinical spectrum of this SCA subtype requires a comprehensive characterization of the structural and functional brain changes in affected individuals. However, due to the rarity of SCA10, limited reports have investigated these changes compared to other types of SCAs (Klockgether et al., 2019).

Structural studies in SCA10 patients have revealed extensive cerebellar atrophy in both white and gray matter, along with moderate cortical, pallidal, brainstem, thalamic, and putaminal degeneration (Arruda et al., 2020; Rasmussen et al., 2001). While initial studies have provided anatomical insights into brain atrophy in SCA10, there is no information regarding functional connectivity (FC) alterations and their possible relationship to clinical metrics.

In this study, we hypothesize that gray matter atrophy contributes directly to alterations in functional connectivity, thereby underscoring the structural–functional deterioration observed in SCA10 patients. Based on this hypothesis, our objectives were to: (1) investigate the FC changes of resting-state networks (RSNs) associated with gray matter atrophy nodes. (2) explore potential alterations in the FC of distinctive RSNs, and (3) determine correlations between the Blood Oxygen Level Dependent (BOLD) signal of both atrophy-based and distinctive RSNs and cognitive and ataxia assessments. By addressing these objectives, we seek to provide a deeper understanding of the neural mechanisms underlying SCA10 and their relationship to clinical manifestations, ultimately contributing to improved diagnostic and therapeutic strategies for this challenging condition.

## Methods

### Participants

Twenty-six Mexican patients of SCA10 were included in this study of whom three were asymptomatic individuals with molecular diagnosis confirming their condition. Inclusion criteria included (1) Patients with a molecular diagnosis of SCA10, and (2) must be at least 18 years of age. Exclusion criteria included (1) Patients with previous brain diseases, such as stroke, (2) Lack of signed informed consent, and (3) Contraindications to MRI scanning. Twenty-six right-handed subjects were initially selected as controls, matched to the SCA10 group for age, sex, and level of education (see Table 1). After a quality check of the rsfMRI data, seven SCA10 participants were excluded. Consequently, the final sample for the rsfMRI analysis included 19 SCA10 patients, who were compared to 20 control subjects. All procedures were approved by the ethics committee of the Universidad Nacional Autónoma de México (UNAM) following the principles outlined in the Helsinki Declaration. Before participation, written consent was obtained from each participant.

**Table 1:** Demographic and clinical characteristics of the participants

	All			fMRI		
	Control	SCA10	*p*-value/t-value	Control	SCA10	*p*-value/stat-value
n	26	26	–	20	19	–
Male/Female	11/15	11/15	χ2 = 0	7/13	0/12	0.90/χ2 = 0.01
Age	50.65 ± 9.28	50.38 ± 9.91	0.922/ t = 0.098	50.56 ± 8.13	50.10 ± 9.8	0.87/ t = 0.15
Year of education	10.15 ± 6	9.73 ± 2.50	0.742/ t = 0.331	9.05 ± 6.13	10.11 ± 2.58	0.49/ t = 0.69
Age of onset	–	33.82 ± 8.66	–	–	34.62 ± 8.56	–
Disease duration	–	16.17 ± 9.57	–	–	14.87 ± 10.34	–
MoCA	27.0 ± 2.42	21.9 ± 4.15	0.001***/ t = 4.27	26.64 ± 2.58	21.3 ± 4.27	0.0009***/ t = 3.70
SARA	–	15.7 ± 7.97	–	–	14.4 ± 8.13	–
Ataxia Severity						
No ataxia = 0		*n* = 0 (0%)			*n* = 0 (0%)	
Mild ataxia = 1–10		*n* = 5 (20%) 3.7 ± 3.4			*n* = 5 (26%) 3.7 ± 3.4	
Moderate ataxia = 11–20		*n* = 13 (50%) 14.9 ± 3.3			*n* = 9 (48%) 15.2 ± 3.0	
Severe ataxia = 21–30		n = (30%) 24.3 ± 3.4			*n* = 5 (26%) 23.5 ± 3.4	
Very severe ataxia = 31–40		*n* = 0 (0%)			*n* = 0 (0%)	

*Values represent mean ± SD

## Clinical assessment

### Severity of Ataxia

The Scale for the Assessment and Rating of Ataxia (SARA) was utilized to evaluate the fundamental symptoms of ataxia in the participants. This concise clinical score consists of 8 items assessing various aspects including gait, stance, sitting, speech disturbance, finger-chase, nose-finger test, fast alternating hand movements, and heel-shin slide. Scores on the SARA range up to a maximum of 40 points, with higher scores indicating more severe ataxia (Schmitz-Hübsch et al., 2006). Additionally, patients were categorized based on their SARA scores denoting ataxia severity. The subgroups were defined as follows: no ataxia (0 points), mild ataxia (1–10 points), moderate ataxia (11–20 points), severe ataxia (21–30 points), and very severe ataxia (31–40 points) (Kim et al., 2011).

### Cognitive Screening

Additionally, the Montreal Cognitive Assessment (MoCA) Spanish version 1 was used as a cognitive screening measure. This assessment evaluates executive function, verbal memory, visuospatial ability, attention, working memory, language, abstract reasoning, and orientation to time and place. The MoCA total score, with a maximum of 30 points, provides an overall assessment of cognitive performance. A score equal to or below 25 points is indicative of mild cognitive impairment (Aguilar-Navarro et al., 2018; Larner, 2016).

## Image acquisition

The imaging procedures were conducted at the Instituto de Neurobiologia of UNAM in Juriquilla, Queretaro, Mexico, utilizing a 3 T General Electric MR750 Discovery scanner equipped with a 32-channel head coil. A high-resolution T1-3D volume was acquired with a TR/TE of 3.18/8.16 ms, flip angle of 9°, and FOV and matrix of 256 × 256, resulting in an isometric resolution of 1 × 1 × 1 mm³. Resting-state functional Magnetic Resonance Imaging (rsfMRI) were obtained using an Echo Planar Imaging single-shot sequence with a TR/TE of 2000/35 ms, FOV of 212 × 212 mm², flip angle of 80°, and 150 whole-brain volumes comprising 91 slices each. The final resolution was 3 × 3 × 4 mm, with no gaps between slices.

## Voxel-based morphometry analysis to determine structural atrophy nodes

The analysis utilized whole-brain Voxel-based Morphometry (VBM) to identify changes in gray matter (GM) volume between groups. FSL-VBM (https://fsl.fmrib.ox.ac.uk/fsl/docs/#/structural/fslvbm) was employed, which includes skull stripping, segmentation into gray matter (GM), white matter, and cerebrospinal fluid. Subsequently, GM images were normalized to MNI152 standard space, modulated, and smoothed with a 3-mm full-width-at-half-maximum Gaussian kernel. Anatomical descriptions of GM changes were referenced using the Harvard-Oxford Cortical Structural Atlas, the Harvard-Oxford Subcortical Structural Atlas, and the Cerebellar Atlas in MNI152. Following this analysis, regions of interest (ROIs) were defined based on the peak local maxima within the significant clusters. For each cluster, the voxels showing the highest statistical difference (t-value > 6) were identified, and a spherical ROI (12-mm radius) was centered on that local maximum.

## Resting-state functional magnetic resonance imaging analysis

The preprocessing of functional images involved several steps: motion and slice timing correction, removal of non-brain tissue, spatial smoothing using a 5-mm full-width-at-half-maximum Gaussian kernel (1.5 times the voxel size), and high-pass temporal filtering equivalent to 100 s (0.01 Hz), in order to remove slow drifts. Also, the resting state signal is low frequency, mostly between 0.01 and 0.1 Hz, consequently, we want to remove frequencies only below 0.01 Hz (corresponding to a period of 100 s). Additionally, we relied on previous research for preprocessing rsfMRI data to identify intrinsic brain activity (Poudel et al., 2014; Werner et al., 2014). Following preprocessing, the resting-state volumes were registered first to the subject’s high-resolution T1-weighted scan and then to the standard MNI152 space using nonlinear registration. Subsequently, ICA-AROMA was applied to effectively eliminate motion-related spurious noise from the rsfMRI datasets (Pruim et al., 2015). After preprocessing, seven participants were discarded due to motion artifacts across volumes that affected subsequent analysis. The final sample size for the rsfMRI analysis included 19 SCA10 patients, who were compared to 20 Control subjects.

### Independent components analysis and dual regression

Independent Components Analysis (ICA) was conducted using MELODIC (Multivariate Exploratory Linear Decomposition into Independent Components) within FSL. Preprocessed volumes were initially analyzed on a single-subject basis. Twenty-five components were extracted per subject, and manual classification of these components was conducted. Components corresponding to resting-state networks (RSNs) were selected for further analysis, while components identified as structured noise were removed through regression-based denoising. The selected RSNs from each subject were then analyzed and grouped using a temporal-concatenation approach, which was based on the frequency spectra of the components’ time courses. The compiled dataset was ultimately decomposed into 25 independent components (Kairov et al., 2017). To identify group RSNs in this study, single ICs were projected on the resting state functional network atlas (available at http://www.fmrib.ox.ac.uk/analysis/royalsoc8/) and visual inspections of previously reported RSNs were performed (Beckmann et al., 2005; Lee et al., 2013; Raichle, 2010; Robinson et al., 2009; Schimmelpfennig et al., 2023; Smith et al., 2009).

To compare RSNs between groups, a dual-regression analysis was employed (Beckmann et al., 2009). This method utilizes the ICs maps as network templates to identify corresponding functional connectivity maps for each subject (Nickerson et al., 2017), integrating temporal information in the rsfMRI data across multiple distributed networks identified in the initial group ICA (Beckmann et al., 2009).

## Seed-based functional connectivity analysis

Four seeds-ROI were defined from the VBM analysis. One seed was demarcated for in the Left Precentral gyrus and three seed in Right Cerebellum VIIIb, Right Cerebellum VI and Left Cerebellum V. A voxel-wise seed-based analysis was conducted by computing Pearson’s correlation between the 4D denoised data and each ROI. The correlation values were then normalized into Fisher’s Z-score to obtain functional maps for each ROI.

### Statistical analysis

Spearman’s partial correlations were calculated between the average BOLD signal of all clusters within each RSN that showed significant differences between groups and the MoCA and SARA scores, controlled by age. This correlation analysis included RSNs obtained from both seed-ROI and ICA approaches. Subsequently, an FDR correction for multiple comparisons was applied, setting a significant threshold of *p* < 0.05. Group comparison for VBM, ICA-Dual regression and Seed-based functional connectivity, between SCA10 patients and Control subjects, were performed using a two tailed two-sample t-test with 10,000 permutations using the general linear model implemented in FSL. Age was included as a nuisance regressor in the analysis. The maps were thresholded at *p* < 0.05 and corrected by Threshold-Free Cluster Enhancement (TFCE) for family-wise error correction. The anatomical descriptions of VBM, ICA and seed-based FC were based on the Harvard-Oxford cortical and subcortical structural atlases and the cerebellar atlas (Diedrichsen et al., 2009). All statistical analyses were performed with R version 4.4.0.

## Results

Demographic data did not show a significant difference between SCA10 and Control subjects for variables such as sex, age, and years of education. In addition, there were no significant differences for the same variables for the subsample of participants included in the rsfMRI analysis (Table 1). However, significant differences were found for MoCA scores considering the whole and partial sample, as reported previously (Chirino-Pérez et al., 2021).

### Voxel-based morphometry analysis

**Fig. 1:**
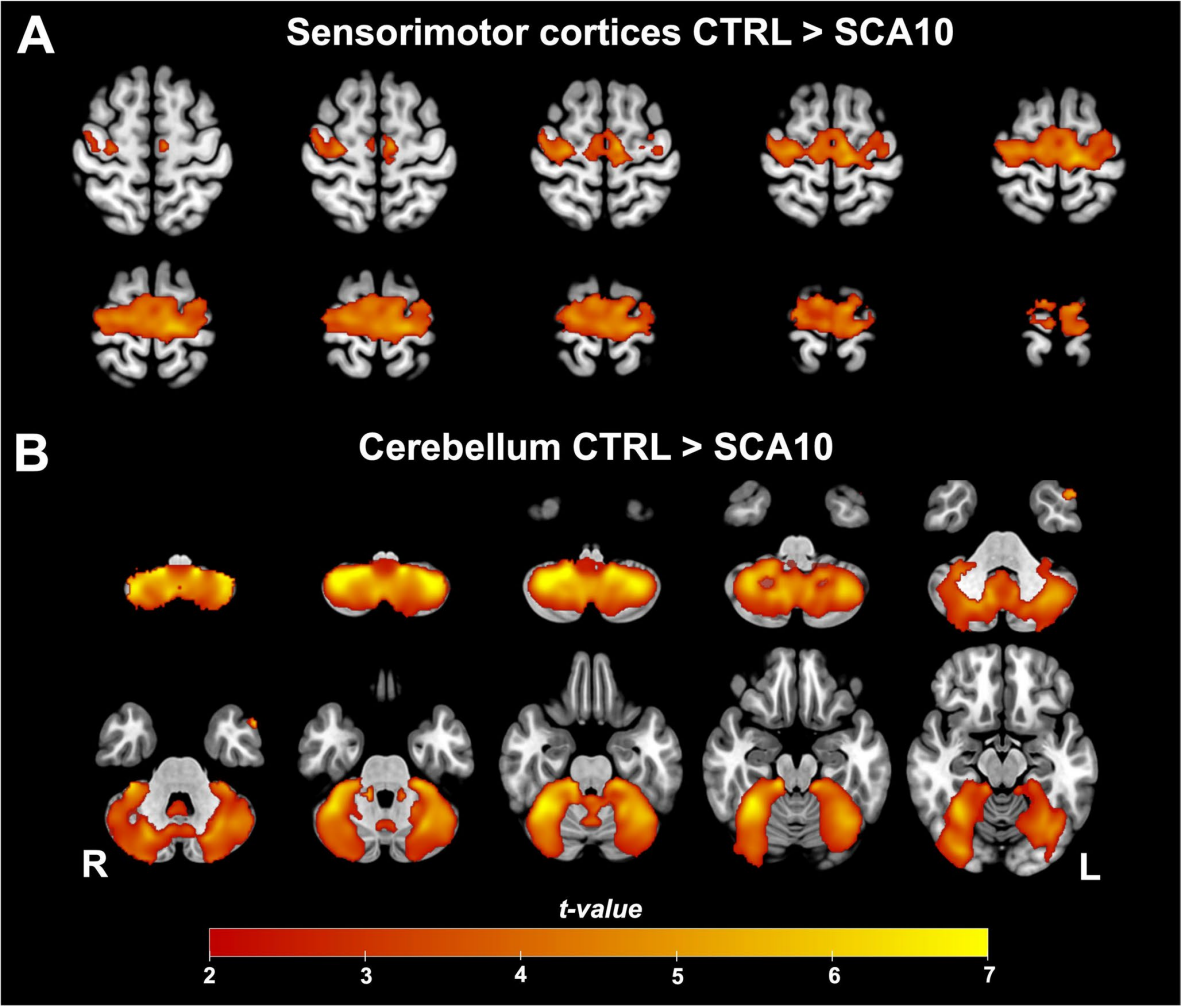
Gray matter Volume reduction in SCA10 patients. Two main clusters were found in the sensorimotor (**A**) cortices and the cerebellum (**B**). Warm colors show the areas where SCA10 patients’ GM decreased significantly compared to Controls (*p* < 0.05)

VBM analysis was performed to determine the GM degeneration in the brain and cerebellum in SCA10 patients. SCA10 patients showed a GM decrease in the cortical mantle encompassing the bilateral precentral gyrus, bilateral postcentral gyrus, bilateral supplementary motor area, temporal pole, and partially the superior frontal gyrus, occipital fusiform gyrus, and the temporal occipital fusiform cortex as well as over the whole cerebellum, especially regions such as bilateral Crus I, Crus II, V, VI, VIIIa, VIIb, VIIIb, and right IX (Fig. 1).

### ROIs-based analysis

**Fig. 2:**
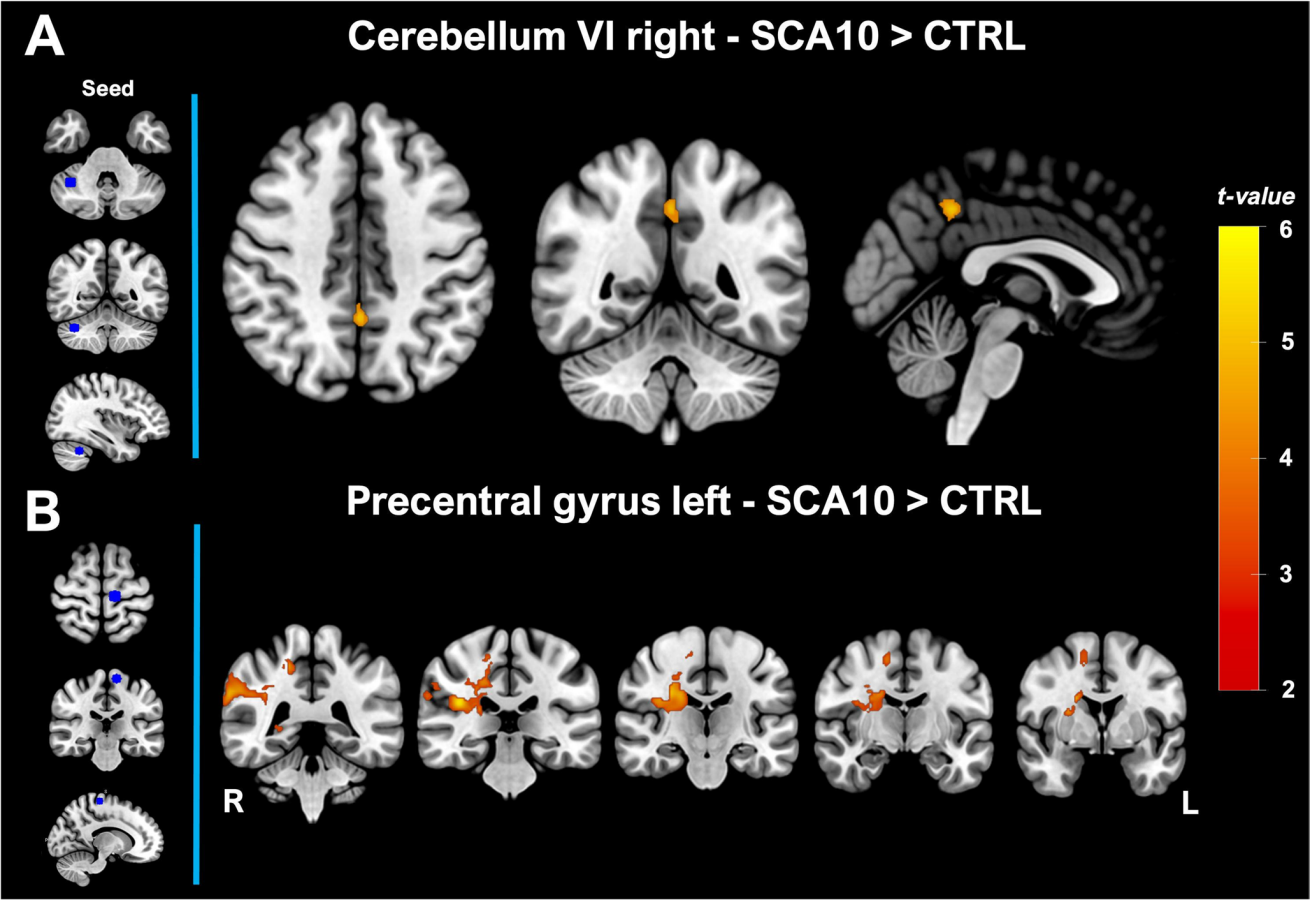
Functional connectivity of seed atrophy based in SCA10 patients. (**A**) shows the FC change for the seed in the cerebellum VI right. (**B**) shows the FC change for the seed in the precentral gyrus left. The left vertical panel showed the location of the spheric seed in blue. The red color bar represents the significant increment of FC in patients with SCA10 (*p* < 0.05)

VBM found two large clusters displaying GM decreases (Table 2). Four spheric ROIs were extracted from these two clusters based on the highest local maxima within them. Regarding the ROIs from the cerebellum, SCA10 patients showed increased FC between right cerebellar VI with precuneus cortex and posterior division of the cingulate gyrus (Fig. 2A). For the seeds located in the right Cerebellum VIIIb and left Cerebellum V no significant differences were found. For the seed located in the left precentral gyrus, significant FC changes were found with the parietal operculum cortex, right precentral and postcentral gyrus, supramarginal gyrus (anterior and posterior division), angular gyrus, precuneus cortex, planum temporalis, parietal operculum, and caudate (Fig. 2B).

**Table 2:** Local maxima coordinate of the seed-ROIs

Anatomical Region	MNI coordinates (voxels)		
	X	Y	Z
Cerebellum			
Right Cerebellum VIIIb	36	40	11
Right Cerebellum VI	27	37	19
Left Cerebellum V	57	46	22
**Brain**			
Left Precentral gyrus	51	50	70

### Independent components analysis

**Fig. 3:**
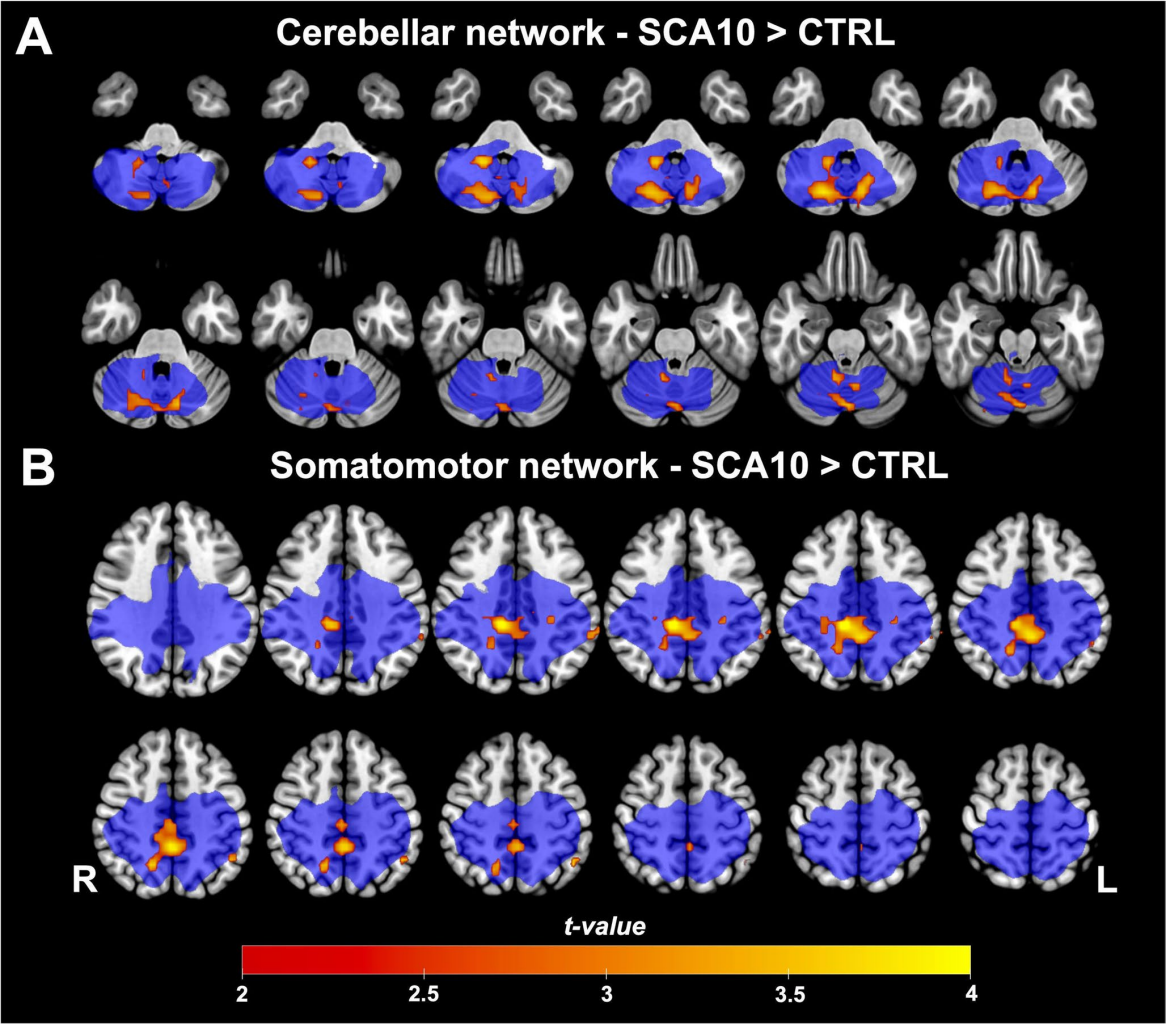
Group differences in cerebellar and somatomotor functional networks. (**A**) Cerebellar Network and (**B**) Somatomotor Network compared to Controls. Warm colors represent where SCA10 patients showed significantly increased functional connectivity within the network (*p* < 0.05. The blue color represents the size and location of the original functional network

Besides the seed-ROI-based FC analyses, we also explored possible FC changes using ICA. Sixteen resting-state functional networks were identified encompassing default mode network, executive control network, medial visual network, lateral visual network, occipital visual network, somatosensory network, superior frontal network, somatosensory motor network, auditory network, executive frontal network, frontoparietal network, salience network, cerebellar network, basal ganglia network and prefrontal network (See supplementary data, Fig. 1). Group comparison between these resting state networks showed that SCA10 patients had higher FC in the sensorimotor network and cerebellar network. Especially, the FC increased in the cerebellar network and was grouped in two clusters located in the bilateral Crus I, Crus II, I-IV, V, VI, VIIb, Vermis VI, and Vermis Crus II. In addition, the sensorimotor network showed an FC increase in three clusters located in the posterior division of the cingulate gyrus, precuneus cortex, precentral gyrus, postcentral gyrus, anterior and posterior division of the supramarginal gyrus, angular gyrus, and superior division of the lateral occipital cortex (Fig. 3).

### Correlation analysis

**Fig. 4:**
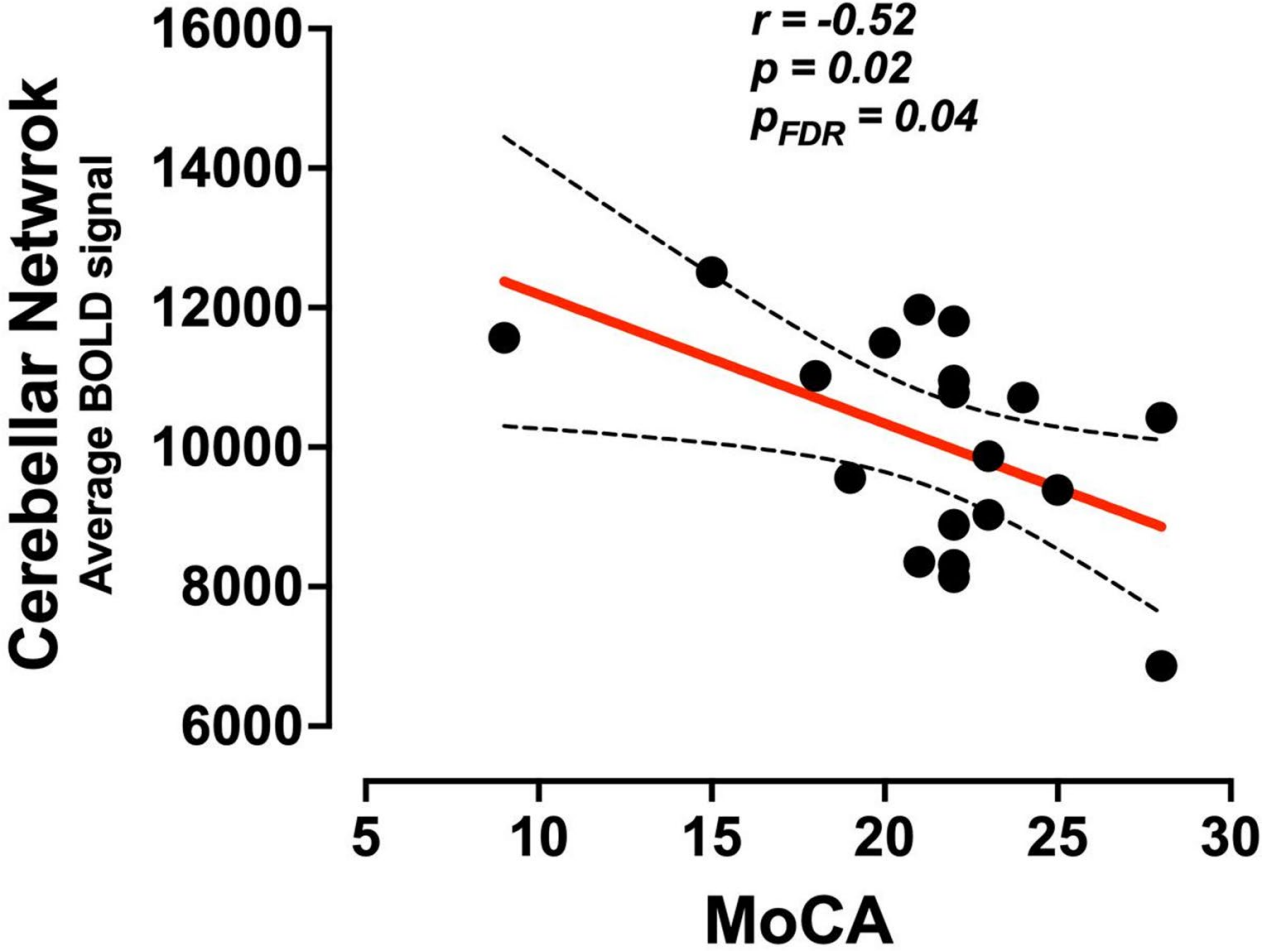
Partial correlation between the Cerebellar network and MoCA scores. Spearman partial correlation between the BOLD signal of the cerebellar network and the MoCA scores (*p* < 0.05)

The partial correlation analysis of the BOLD signal of the significant RSNs, obtained from both the seed-ROI and ICA analysis, showed that only the Cerebellar network had a significant negative relationship with MoCA scores; *r* = −0.52, *p* = 0.02, p-FDR = 0.04 (Fig. 4). There were no significant correlations between any BOLD signal of RSNs and SARA scores.

## Discussion

SCA10 is a neurodegenerative disease characterized by structural and functional brain degeneration. Using multimodal MRI, we explored cerebral and cerebellar gray matter atrophy and functional connectivity changes in RSNs associated with GM atrophy. We identified bilateral GM reduction in the cerebellum and motor cortices. The most significant peaks within these atrophy clusters were located in the right cerebellum VI and the left precentral gyrus. Additionally, these ROIs showed higher FC changes in SCA10 patients. Moreover, the canonical cerebellar and somatomotor functional networks exhibited increased FC connectivity in SCA10 patients as well. Lastly, the BOLD signal of the cerebellar network correlated negatively with the cognitive MoCA performance in these patients. These findings provide a comprehensive characterization of alterations in spontaneous resting state networks linked to gray matter atrophy patterns in SCA10 patients. This study represents a founding functional description of SCA10, offering insights into the neurobiological underpinnings of the disease.

SCA10 is a genetic disease primarily affecting the cerebellum. In our study, we found that the cerebellum and the motor and sensorimotor cortices were the two main affected regions. Previous studies in the Brazilian SCA10 population showed extensive cerebellar atrophy along with pallidal reduction (Arruda et al., 2020). Cortical thickness reduction was observed in the left frontal (frontal pole, orbital middle frontal, and rostral anterior cingulate), temporal (bilateral parahippocampal gyrus), and left occipital (lingual gyrus) lobes. In previous research, with a similar sample size in a Mexican population, the atrophy pattern included the cerebellum, brain stem, thalamus, putamen, and cingulate and precentral gyrus (Hernandez-Castillo et al., 2019). Despite slight differences in atrophy patterns, GM reduction cores remain in the same anatomical areas: the cerebellum and sensorimotor cortices.

In SCA10 patients, we identified an atrophy core located in the right cerebellum VI, which exhibited higher functional connectivity with the precuneus cortex and the posterior division of the cingulate gyrus. The precuneus is critically involved in higher-order cognitive processes such as visuospatial imagery, episodic memory retrieval, and self-processing operations, functional imaging studies indicate that precuneus activation is associated with both autobiographical/contextual memory recall and imagery-related aspects of episodic retrieval (Cavanna & Trimble, 2006), while the posterior division of the cingulate gyrus is associated with emotional regulation and episodic memory processing, particularly the integration of autobiographical and internally directed information. This increased connectivity may suggest a compensatory mechanism within the brain’s networks due to cerebellar degeneration; thus, this may reflect both its centrality in cerebro-cerebellar networks and its susceptibility to disease-related disruption in SCA10. Additionally, the precuneus and posterior division of the cingulate gyrus are key nodes within the DMN, which is active during rest and involved in internally directed cognitive functions. In our study, we did not find any significant changes in the DMN network as has been reported in SCA3 (Guo et al., 2023), which represents that the functional integrity of this network remains unaffected at this point in these patients. Additionally, the observed higher functional connectivity between the cerebellum and cingulate gyrus may reflect the cerebellum’s broader role in integrating cognitive and emotional information (Rudolph et al., 2023).

A comparison of canonical spontaneous resting state networks showed higher FC in the sensorimotor network. This greater FC may imply an enhanced interaction between motor and sensory processing areas, possibly as a response to cerebellar dysfunction. On the other hand, the higher FC displayed in the cerebellar networks may suggest extensive involvement of the cerebellum in compensatory mechanisms or network reorganization in response to disease-related atrophy as has been shown in SCA2 (Siciliano et al., 2023).

These findings indicate that SCA10 not only affects motor coordination but also impacts broader networks associated with sensory processing and cognitive functions. The increased connectivity within these networks may reflect a compensatory process aimed at maintaining functional integrity despite ongoing neurodegeneration. These findings provide valuable insights into the neural mechanisms underlying motor impairments associated with SCA10, suggesting a disrupted interplay between the cerebellum and motor-related brain regions (Chirino-Pérez et al., 2021).

Finally, we found a correlation between cerebellar network functional connectivity and patients’ total MoCA scores. Although the cerebellum has traditionally been regarded as a regulator of motor actions, increasing evidence indicates that it also plays a central role in cognitive and affective functions. In our study, higher cerebellar connectivity was associated with lower MoCA scores, a counterintuitive finding that may reflect maladaptive network reorganization that interfere with cognitive performance. Similar associations have been reported in other spinocerebellar ataxias. In SCA6, Pereira et al. (2017) described an anticorrelation between global network connectivity and cognitive performance. In SCA2, Hernandez-Castillo et al. (2015) observed increased default-mode connectivity correlating with longer reaction times (i.e., worse performance). In Friedreich’s ataxia, (Cocozza et al., 2018 found altered connectivity related to clinical variables, although these correlations did not survive correction for multiple comparisons; however, the directionality of the associations paralleled those observed in SCA. In contrast, Guo et al. (2023) reported in SCA3 a more expected positive association, with disrupted DMN–frontoparietal connectivity linked to lower MMSE scores. These findings suggest that both hypo- and hyperconnectivity can emerge in the context of cerebellar degeneration, and that hyperconnectivity may sometimes represent maladaptive compensation rather than efficient reorganization. Although the MoCA is a screening tool and our results should be interpreted with caution, they underscore the potential contribution of the cerebellum to cognitive functioning. More detailed neuropsychological assessments will be necessary to delineate the specific domains involved, in line with previous evidence linking visuospatial memory and executive impairments in SCA10 patients to the posterior cerebellum, as well as prefrontal, cingulate, and middle temporal cortices (Chirino-Pérez et al., 2021). However, further analyses are needed to fully understand these changes and their potential consequences on the motor and cognitive performance of the patients. To gain a more comprehensive understanding of cognitive alterations in SCA10 patients, incorporating more specific assessments is crucial. The Schmahmann Scale (Hoche et al., 2018), which is specifically designed for the assessment of cerebellar cognitive affective syndrome (CCAS) could provide deeper insights into the specific cognitive deficits associated with cerebellar dysfunction in SCA10, to evaluate executive, linguistic, visuospatial and affective impairments.

### Limitations and future research directions

This study has several limitations that warrant consideration. First, the relatively small sample size obtained after rsfMRI quality control reduces the generalizability of our findings and may not fully represent the spectrum of disease severity in SCA10. Motion artifacts were particularly problematic, leading to the exclusion of severely affected patients, which underscores the need for stricter motion-control strategies in future studies to minimize data loss and preserve sample representativeness. Second, our rsfMRI acquisition was relatively short, limiting the ability to capture the full dynamics of the BOLD signal. Although practical constraints such as scan duration, participant tolerance, and cost-effectiveness must be carefully balanced in clinical populations, longer acquisitions of 10–15 min are increasingly considered the current best practice for reliable functional connectivity analysis. Therefore, to improve sensitivity and reproducibility extended rsfMRI acquisition may be implemented. Future research will address these limitations by implementing a longitudinal design, enabling the assessment of both disease progression and functional connectivity changes over time. Beyond seed-based approaches, alternative analytic strategies can be applied, with a particular emphasis on cerebellar connectivity, since data-driven methods such as ICA may overlook disease-relevant changes in this region. Increasing the sample size remains a priority to enhance statistical power and achieve better representation across the full clinical spectrum. Additionally, we intend to explore sex-related differences, which may provide further insight into the heterogeneity of SCA10. Collectively, these methodological refinements will allow us to more comprehensively characterize the structural–functional relationships underlying SCA10 and strengthen the translational value of our findings.

## Conclusion

In conclusion, our study provides new insights into the neurobiological mechanisms of SCA10 by demonstrating network-level functional connectivity alterations associated with cerebellar atrophy. We identified widespread grey matter degeneration in motor cortices and the cerebellum, accompanied by reduced functional connectivity within somatomotor and cerebellar networks, indicating closely coupled structural–functional deterioration. Notably, cerebellar network connectivity showed a negative correlation with MoCA scores, suggesting maladaptive compensation rather than efficient reorganization. Together, these findings underscore the impact of SCA10-related degeneration on resting-state networks and point toward maladaptive functional connectivity changes as a potential mechanism contributing to cognitive and motor dysfunction.

## Supplementary Information

Below is the link to the electronic supplementary material.

Supplementary Material 1 (DOCX 1.08 MB)

## Data Availability

The data and materials supporting the findings of this study are available from the corresponding author upon reasonable request. In addition, a testing script to perform all MRI analysis implemented in our study has been deposited at the following link: [https://github.com/jokasta57/dualRegression\_rois\_fMRI ](https:/github.com/jokasta57/dualRegression_rois_fMRI).

## References

[CR1] Aguilar-Navarro, S. G., Mimenza-Alvarado, A. J., Palacios-García, A. A., Samudio-Cruz, A., Gutiérrez-Gutiérrez, L. A., & Ávila-Funes, J. A. (2018). Validity and reliability of the Spanish version of the Montreal Cognitive Assessment (MoCA) for the detection of cognitive impairment in Mexico. *Revista Colombiana De Psiquiatría,**47*(4), 237–243. 10.1016/j.rcp.2017.05.003 30286846 10.1016/j.rcp.2017.05.003

[CR2] Arruda, W. O., Meira, A. T., Ono, S. E., de Carvalho Neto, A., Betting, L. E. G. G., Raskin, S., et al. (2020). Volumetric MRI changes in spinocerebellar ataxia (SCA3 and SCA10) patients. *The Cerebellum,**19*(4), 536–543. 10.1007/s12311-020-01137-3 32367276 10.1007/s12311-020-01137-3

[CR3] Beckmann, C. F., DeLuca, M., Devlin, J. T., & Smith, S. M. (2005). Investigations into resting-state connectivity using independent component analysis. *Philosophical Transactions of the Royal Society B: Biological Sciences,**360*(1457), 1001–1013. 10.1098/rstb.2005.1634 10.1098/rstb.2005.1634 PMC1854918 16087444

[CR4] Beckmann, C., Mackay, C., Filippini, N., & Smith, S. (2009). Group comparison of resting-state FMRI data using multi-subject ICA and dual regression. *NeuroImage,**47*, Article S148. 10.1016/s1053-8119(09)71511-3

[CR5] Cavanna, A. E., & Trimble, M. R. (2006). The precuneus: A review of its functional anatomy and behavioural correlates. *Brain,**129*(3), 564–583. 10.1093/brain/awl004 16399806 10.1093/brain/awl004

[CR6] Chirino-Pérez, A., Vaca-Palomares, I., Torres, D. L., Hernandez-Castillo, C. R., Diaz, R., Ramirez-Garcia, G., & Fernandez-Ruiz, J. (2021). Cognitive impairments in spinocerebellar ataxia type 10 and their relation to cortical thickness. *Movement Disorders,**36*(12), 2910–2921. 10.1002/mds.28728 34327752 10.1002/mds.28728

[CR7] Cocozza, S., Costabile, T., Tedeschi, E., Abate, F., Russo, C., Liguori, A., et al. (2018). Cognitive and functional connectivity alterations in Friedreich’s ataxia. *Annals of Clinical and Translational Neurology,**5*(6), 677–686. 10.1002/acn3.555 29928651 10.1002/acn3.555 PMC5989773

[CR8] Diedrichsen, J., Balsters, J. H., Flavell, J., Cussans, E., & Ramnani, N. (2009). A probabilistic MR atlas of the human cerebellum. *NeuroImage,**46*(1), 39–46. 10.1016/j.neuroimage.2009.01.045 19457380 10.1016/j.neuroimage.2009.01.045

[CR9] Gatto, E. M., Gao, R., White, M. C., Uribe Roca, M. C., Etcheverry, J. L., Persi, G., et al. (2007). Ethnic origin and extrapyramidal signs in an Argentinean spinocerebellar ataxia type 10 family. *Neurology,**69*(2), 216–218. 10.1212/01.wnl.0000265596.72492.89 17620556 10.1212/01.wnl.0000265596.72492.89

[CR10] Guo, J., Jiang, Z., Liu, X., Li, H., Biswal, B. B., Zhou, B., et al. (2023). Cerebello-cerebral resting-state functional connectivity in spinocerebellar ataxia type 3. *Human Brain Mapping*, *44*(3), 927–936. 10.1002/hbm.26113 36250694 10.1002/hbm.26113 PMC9875927

[CR11] Hernandez-Castillo, C. R., Diaz, R., Vaca-Palomares, I., Torres, D. L., Chirino, A., Campos-Romo, A., et al. (2019). Extensive cerebellar and thalamic degeneration in spinocerebellar ataxia type 10. *Parkinsonism & Related Disorders,**66*, 182–188. 10.1016/j.parkreldis.2019.08.011 31445906 10.1016/j.parkreldis.2019.08.011

[CR12] Hoche, F., Guell, X., Vangel, M. G., Sherman, J. C., & Schmahmann, J. D. (2018). The cerebellar cognitive affective/Schmahmann syndrome scale. *Brain,**141*(1), 248–270. 10.1093/brain/awx317 29206893 10.1093/brain/awx317 PMC5837248

[CR13] Kairov, U., Cantini, L., Greco, A., Molkenov, A., Czerwinska, U., Barillot, E., & Zinovyev, A. (2017). Determining the optimal number of independent components for reproducible transcriptomic data analysis. *BMC Genomics,**18*(1), 1–13. 10.1186/s12864-017-4112-9 28893186 10.1186/s12864-017-4112-9 PMC5594474

[CR14] Kim, B.-R., Lim, J.-H., Lee, S. A., Park, S., Koh, S.-E., Lee, I.-S., et al. (2011). Usefulness of the scale for the assessment and rating of ataxia (SARA) in Ataxic Stroke Patients. *Annals of Rehabilitation Medicine,**35*(6), Article 772. 10.5535/arm.2011.35.6.772 22506205 10.5535/arm.2011.35.6.772 PMC3309386

[CR15] Klockgether, T., Mariotti, C., & Paulson, H. L. (2019). Spinocerebellar ataxia. *Nature Reviews. Disease Primers,**5*(1), 1–21. 10.1038/s41572-019-0074-3 10.1038/s41572-019-0074-3 30975995

[CR16] Larner, A. J. (2016). Cognitive screening instruments: A practical approach. *Cognitive Screening Instruments: A Practical Approach*. 10.1007/978-3-319-44775-9

[CR17] Lee, M. H., Smyser, C. D., & Shimony, J. S. (2013). Resting-state fMRI: A review of methods and clinical applications. *American Journal of Neuroradiology,**34*(10), 1866–1872. 10.3174/ajnr.A3263 22936095 10.3174/ajnr.A3263 PMC4035703

[CR18] Matsuura, T., Yamagata, T., Burgess, D. L., Rasmussen, A., Grewal, R. P., Watase, K., et al. (2000). Large expansion of the ATTCT pentanucleotide repeat in spinocerebellar ataxia type 10. *Nature Genetics,**26*(2), 191–194. 10.1038/79911 11017075 10.1038/79911

[CR19] Nickerson, L. D., Smith, S. M., Öngür, D., & Beckmann, C. F. (2017). Using dual regression to investigate network shape and amplitude in functional connectivity analyses. *Frontiers in Neuroscience,**11*(MAR), 1–18. 10.3389/fnins.2017.00115 28348512 10.3389/fnins.2017.00115 PMC5346569

[CR20] Pereira, L., Airan, R. D., Fishman, A., Pillai, J. J., Kansal, K., Onyike, C. U., et al. (2017). Resting-state functional connectivity and cognitive dysfunction correlations in spinocerebelellar ataxia type 6 (SCA6). *Human Brain Mapping,**38*(6), 3001–3010. 10.1002/hbm.23568 28295805 10.1002/hbm.23568 PMC6866919

[CR21] Poudel, G. R., Egan, G. F., Churchyard, A., Chua, P., Stout, J. C., & Georgiou-Karistianis, N. (2014). Abnormal synchrony of resting state networks in premanifest and symptomatic Huntington disease: The IMAGE-HD study. *Journal of Psychiatry & Neuroscience,**39*(2), 87–96. 10.1503/jpn.120226 24083458 10.1503/jpn.120226 PMC3937285

[CR22] Pruim, R. H. R., Mennes, M., van Rooij, D., Llera, A., Buitelaar, J. K., & Beckmann, C. F. (2015). ICA-AROMA: A robust ICA-based strategy for removing motion artifacts from fMRI data. *NeuroImage,**112*, 267–277. 10.1016/j.neuroimage.2015.02.064 25770991 10.1016/j.neuroimage.2015.02.064

[CR23] Raichle, M. E. (2010). Two views of brain function. *Trends in Cognitive Sciences,**14*(4), 180–190. 10.1016/j.tics.2010.01.008 20206576 10.1016/j.tics.2010.01.008

[CR24] Rasmussen, A., Matsuura, T., Ruano, L., Yescas, P., Ochoa, A., Ashizawa, T., & Alonso, E. (2001). Clinical and genetic analysis of four Mexican families with spinocerebellar ataxia type 10. *Annals of Neurology*, *50*(2), 234–239. 10.1002/ana.1081 11506407 10.1002/ana.1081

[CR25] Robinson, S., Basso, G., Soldati, N., Sailer, U., Jovicich, J., Bruzzone, L., et al. (2009). A resting state network in the motor control circuit of the basal ganglia. *BMC Neuroscience,**10*, 1–14. 10.1186/1471-2202-10-137 19930640 10.1186/1471-2202-10-137 PMC2785820

[CR26] Rudolph, S., Badura, A., Lutzu, S., Pathak, S. S., Thieme, A., Verpeut, J. L. (2023). *Cognitive-Affective Functions of the Cerebellum*, 43(45), 7554–7564. 10.1523/JNEUROSCI.1451-23.2023 PMC10634583 37940582

[CR27] Schimmelpfennig, J., Topczewski, J., Zajkowski, W., & Jankowiak-Siuda, K. (2023). The role of the salience network in cognitive and affective deficits. *Frontiers in Human Neuroscience*, *17*(March), 1–9. 10.3389/fnhum.2023.1133367 10.3389/fnhum.2023.1133367 PMC10067884 37020493

[CR28] Schmitz-Hübsch, T., Montcel, D., Baliko, S. T., Berciano, L., Boesch, J., Depondt, S., C., et al. (2006). Scale for the assessment and rating of ataxia: Development of a new clinical scale. *Neurology*, *66*(11), 1717–1720. 10.1212/01.wnl.0000219042.60538.92 16769946 10.1212/01.wnl.0000219042.60538.92

[CR29] Siciliano, L., Olivito, G., Urbini, N., Silveri, M. C., & Leggio, M. (2023). The rising role of cognitive reserve and associated compensatory brain networks in spinocerebellar ataxia type 2. *Journal of Neurology*, *270*(10), 5071–5084. 10.1007/s00415-023-11855-3 37421466 10.1007/s00415-023-11855-3 PMC10511586

[CR30] Smith, S. M., Fox, P. T., Miller, K. L., Glahn, D. C., Fox, P. M., Mackay, C. E., et al. (2009). Correspondence of the brain’s functional architecture during activation and rest. *Proceedings of the National Academy of Sciences of the United States of America,**106*(31), 13040–13045. 10.1073/pnas.0905267106 19620724 10.1073/pnas.0905267106 PMC2722273

[CR31] Teive, H. A. G., & Arruda, W. O. (2009). Cognitive dysfunction in spinocerebellar ataxias. *Dementia & Neuropsychologia,**3*(3), 180–187. 10.1590/s1980-57642009dn30300002 29213626 10.1590/S1980-57642009DN30300002 PMC5618971

[CR32] Werner, C. J., Dogan, I., Saß, C., Mirzazade, S., Schiefer, J., Shah, N. J., et al. (2014). Altered resting-state connectivity in huntington’s disease. *Human Brain Mapping*, *35*(6), 2582–2593. 10.1002/hbm.22351 23982979 10.1002/hbm.22351 PMC6869508

