# Supplementary material for: Functional connectivity alterations in spinocerebellar ataxia type 10: insights from gray matter atrophy
Source: Brain Imaging Behav. 2026 Feb 7;20(1):3. doi: 10.1007/s11682-026-01091-4 (PMC12882854; doi:10.1007/s11682-026-01091-4)
Supplement: Supplementary file 1 — Supplementary Material 1 (DOCX 1.08 MB) [file 11682_2026_1091_MOESM1_ESM.docx]

**Supplementary data**

**
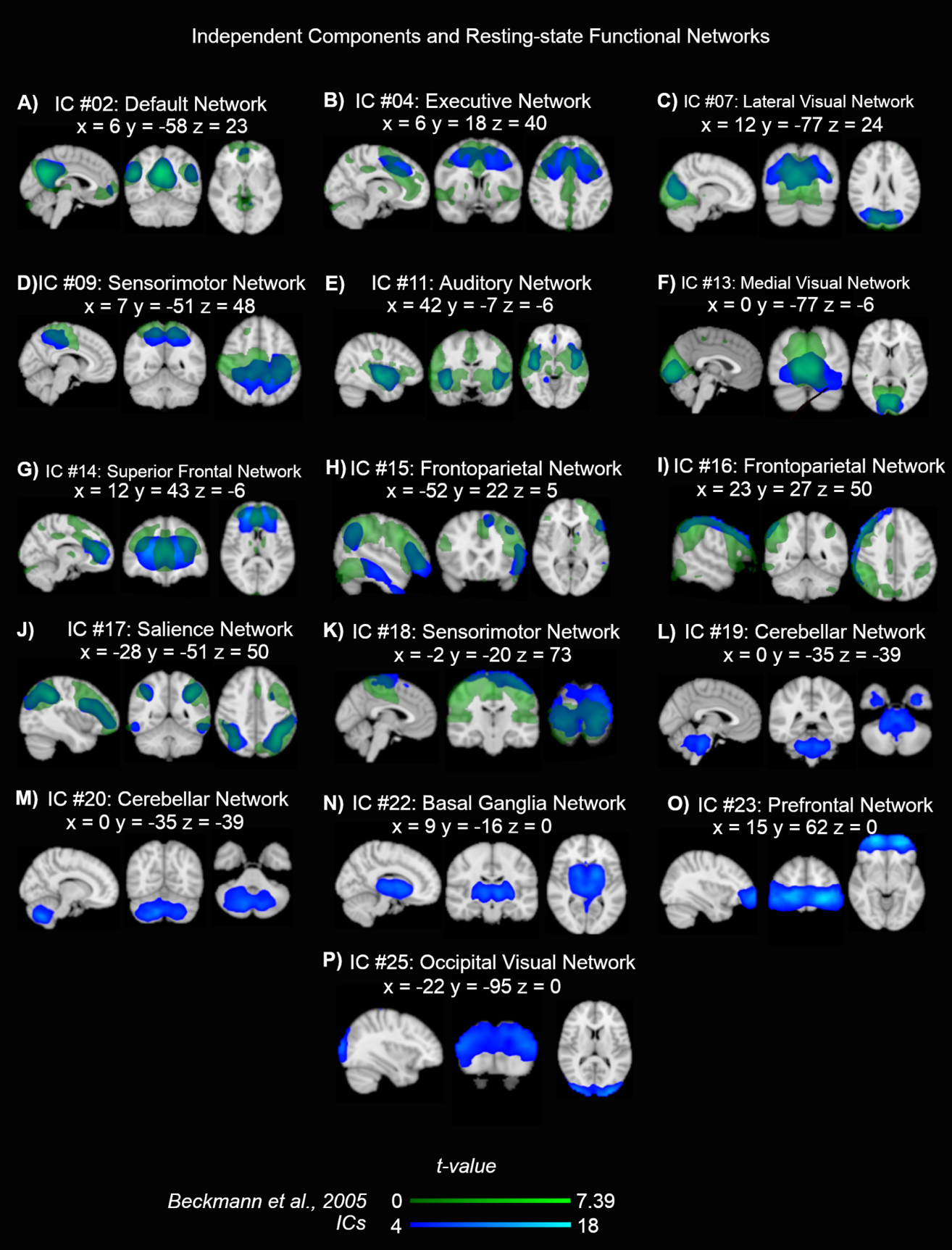
**

**Fig. SI1** Sixteen Functional Networks were identified, after ICA, by overlapping a template of RSNs (blue and green color: A-K), and visual inspection (blue color: A-P)
